# Coming This Way at Last

**Published:** 1902-05

**Authors:** 


### COMING THIS WAY AT LAST.

It is a hopeful sign of the times that at last the *people* are being aroused to the dangers of infectious diseases in sleeping cars and hotels, and so are the railroads. Reference was made in our last to the open letter from the Drummers' Association, asking that sleepers and day coaches be disinfected. We learn that several damage suits against a certain railroad, for infecting passengers, have been decided for plaintiff, and approved by the Supreme Court. The railroads will find it to their interest to sterilize all coaches, and I think will not wait to be compelled by the State Health Officer to do it; and it will be a big thing for the competing roads to advertise "all day and night coaches thoroughly sterilized." Dr. Tabor, the State Health Officer, will cause all roads to carry earthen spittoons and to use bichloride to disinfect them. I understand that Dr. Jameson, Chief Surgeon of the I. & G. N. R. R., will at once institute fumigation of cars.

And the census people are touching up our Governor on registration of vital statistics.

